# Mortality Rates in Staged Bilateral Total Knee Arthroplasty: A Systematic Review and Meta-Analysis

**DOI:** 10.7759/cureus.70736

**Published:** 2024-10-02

**Authors:** Abdullah Krimly, Raed M Sharaf

**Affiliations:** 1 Faculty of Medicine, King Abdulaziz University, Jeddah, SAU

**Keywords:** arthroplasty, knee replacement, mortality, staged bilateral total knee arthroplasty, total knee

## Abstract

This systematic review and meta-analysis aimed to assess the mortality rates of staged bilateral total knee arthroplasty (staBTKA) procedures. This systematic review followed the suggestions and recommendations of the Preferred Reporting Items for Systematic Reviews and Meta-Analyses (PRISMA) 2020. We performed a primary search in the PubMed, EBSCO, Scopus, Web of Science via Clarivate, and Google Scholar databases. A quantitative data synthesis was conducted to estimate the pooled prevalence of mortality among patients who underwent staBTKA using the MedCalc® Statistical Software version 20.115 (MedCalc Software Ltd., Ostend, Belgium). The Newcastle-Ottawa Scale was used for the quality assessment. The study included 29 studies with data from 115,348 patients. The mortality rate was estimated to be 0.34% (95% confidence interval: 0.18% to 0.55%). The data showed significant interstudy heterogeneity (I^2^ = 93.3%). We used funnel plot inspection to visually assess significant publication bias, which showed a symmetrical distribution of the plotted data. Our study found that the mortality rates following staBTKA are relatively high. However, the reliability of our findings is limited due to significant heterogeneity. We recommend that further studies be conducted to provide higher-quality evidence to assess mortality in staBTKA and its trends.

## Introduction and background

The number of total knee arthroplasty (TKA) procedures performed annually has increased dramatically, along with rising life expectancy, a high-demand lifestyle, and remarkable TKA outcomes. In the United States, the total knee replacement rate has increased since 2000, with approximately 12% of the population experiencing knee osteoarthritis (OA). From an economic standpoint, an estimated 10.2 billion US dollars are spent on TKA each year [1]. TKA, with the potential to dramatically reduce pain, restore physical function, and enhance the quality of life, is regarded as the best therapeutic option [2] for patients with end-stage knee arthropathy.

TKA is one of the most popular orthopedic procedures. In the United Kingdom, a total of 99,093 TKA procedures were performed in 2018 [3]. According to current predictions, 1,065,000 primary TKAs would have been performed in the United States in 2020. By 2040, this number is expected to increase to 3,416,000 primary TKAs [4]. Patients with bilateral knee arthritis who require TKA have the choice of having both knees replaced simultaneously (simultaneous bilateral TKA (simBTKA)) or sequentially (staged bilateral TKA (staBTKA)). When given the choice, most patients choose to undergo simBTKA rather than staBTKA, which involves having the operations performed on separate days [5,6]. Over the last 10 years, the number of simBTKAs has steadily increased, with their outcomes documented in several collaborative studies [7-9]. It has been suggested that staBTKA might reduce the risk of complications while increasing the total treatment cost [10-12]. This systematic review and meta-analysis aimed to assess the mortality rates in staBTKA procedures.

## Review

Methods

Study Design

The study was conducted in accordance with the standards and recommendations of the Preferred Reporting Items for Systematic Reviews and Meta-Analyses (PRISMA) 2020 [13].

Literature Search

PubMed, EBSCO, Scopus, Web of Science via Clarivate, and Google Scholar databases were used to compile studies. We combined keywords, Medical Subject Headings (MeSH) phrases, and the Boolean operators AND and OR to perform the database search. Bilateral total knee arthroplasty; Bilateral TKA; Knee replacement; Knee arthroplasty; mortality; death; fatal*; loss; staged; Staged BTKA; and StaBTKA were among the search terms. We conducted a manual Google search in addition to database searches. No language restrictions were applied, and searches for publications from January 1970 and earlier were conducted because TKA was initially documented in the 1970s and 1980s [14,15].

Study Selection and Data Abstraction

Three authors reviewed the titles, abstracts, or full texts of the search results to determine if they matched the criteria for inclusion in this systematic review. Conflicts between two authors were settled through a debate with a third author and by reaching a consensus. The corresponding author was contacted via email if further details of possible research were required. All relevant data from the included articles were extracted and entered into a Microsoft Excel sheet (Microsoft Corp., Redmond, WA, USA).

To manage the primary search results and remove duplicates, search results were retrieved and imported into the Rayyan-Intelligent Systematic Reviews website (Rayyan Systems Inc., Cambridge, MA, USA) [16]. Title, abstract, and full-text screening was performed, and data from the included studies were extracted and entered into a Microsoft Excel sheet.

Risk of Bias Assessment

Two authors evaluated the quality of the listed studies using the Newcastle-Ottawa Scale (NOS) [17]. The values of the quality assessment of the studies ranged from 0 to 9. When a study received seven or more stars, it was deemed to be of good quality.

Data Analysis

The MedCalc® Statistical Software version 20.115 (MedCalc Software Ltd., Ostend, Belgium) was used for quantitative data synthesis. Mortality data were included in a proportion random-effects meta-analysis. Forest and funnel plots were then constructed. The Higgins I^2^ test was used to evaluate the percentage of variability across studies with a significant heterogeneity cutoff point at I^2 ^> 50%. Funnel plots were used to visually assess publication bias with a P-value cutoff point of 0.1 or less.

Results

Search Results

**Figure 1:**
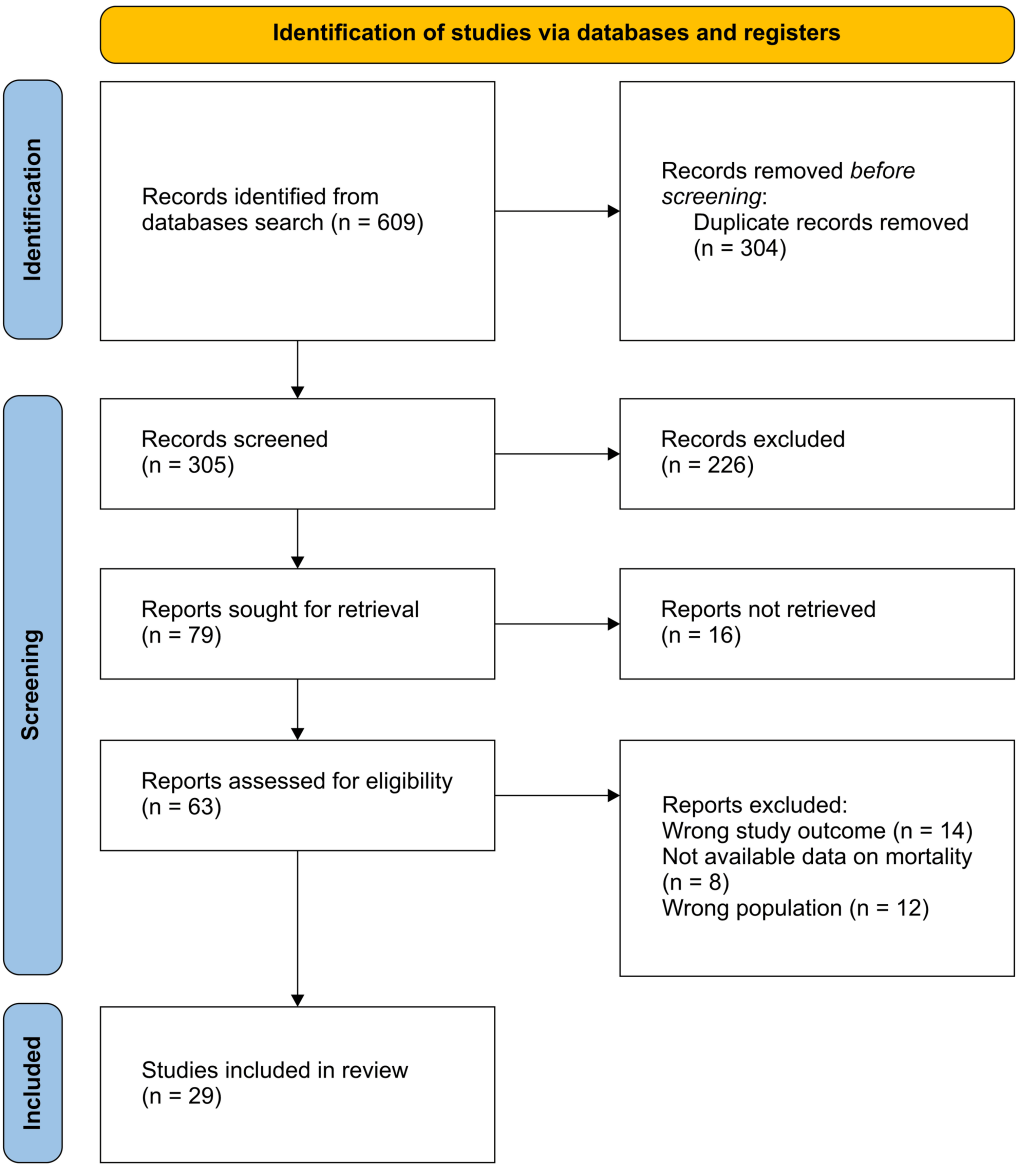
PRISMA flowchart for the summary of the search and screening processes PRISMA: Preferred Reporting Items for Systematic Reviews and Meta-Analyses

We retrieved 609 studies by searching the aforementioned electronic databases. Detection and removal of duplicates resulted in the removal of 304 studies, with 305 studies eligible for title and abstract screening. Following the title and abstract screening, 226 studies were excluded for irrelevant objectives. A full-text assessment of 63 studies was conducted, and 29 studies were included. Figure 1 summarizes the search and study selection processes.

Characteristics of the Included Studies

Twenty-nine studies were finally included in the systematic review. Overall, 115,348 patients (230,696 knees) were enrolled in the pooled analyses [6,18-45] (Table 1). The proportion of men ranged from 4.1% [31] to 51% [25]. The mean age of the population ranged from 61.6 years [24] to 74.1 years [22].

**Table 1:** Characteristics of the included studies NOS: Newcastle-Ottawa Scale

Study	Design	Country	Total population	Males (%)	Mean age (y)	NOS
Berend et al., 2011 [18]	Retrospective	USA	141		62.7	9
Biazzo et al., 2019 [19]	Retrospective	Italy	51	37.30%	68.5	7
Bini et al., 2014 [20]	Retrospective	USA	2123	34.20%	67	6
Bohm et al., 2016 [21]	Retrospective	Canada	25253	39%	66	7
Bolognesi et al., 2013 [22]	Retrospective	USA	3788	39%	74.1	6
Chan et al., 2009 [23]	Retrospective	UK	80	43.80%	66.4	7
Chen et al., 2013 [24]	Prospective	Singapore	47	23.40%	61.6	6
Chua et al., 2018 [25]	Retrospective	Australia	12951	50.60%		7
Courtney et al., 2014 [26]	Retrospective	USA	131	23%	64.2	6
Feng et al., 2019 [27]	Prospective	China	54	9.30%	64.2	7
Forster et al., 2006 [28]	Retrospective	Australia	36	50%	68	7
Gill et al., 2020 [29]	Retrospective	Australia	46	50%	70.7	7
Hutchinson et al., 2006 [30]	Prospective	Australia	125	38%	65	7
Koh et al., 2018 [31]	Retrospective	South Korea	633	4.10%	69.7	8
Lindberg-Larsen et al., 2015 [32]	Retrospective	Denmark	628	42.90%	66	7
Mangaleshkar et al., 2001 [33]	Retrospective	UK	34	38.30%	71.7	6
Meehan et al., 2011 [34]	Retrospective	USA	23715	38.70%	67.7	8
Niki et al., 2014 [35]	Prospective	Japan	60	16.70%	72.3	7
Poultsides et al., 2013 [36]	Retrospective	USA	1151	32.20%	69.5	8
Richardson et al., 2019 [37]	Retrospective	USA	6110			6
Ritter et al., 2003 [6]	Retrospective	USA	152	23%	69.2	6
Sheth et al., 2016 [38]	Retrospective	USA	7185	36.60%	66.8	8
Siedlecki et al., 2018 [39]	Retrospective	France	26	26.90%	70	8
Sliva et al., 2005 [40]	Retrospective	USA	306	34.60%	67.2	6
Stefánsdóttir et al., 2008 [41]	Retrospective	Sweden	3432	37.50%	71.2	7
Tsay et al., 2019 [42]	Retrospective	USA	45419	37.40%	66.6	8
Walmsley et al., 2006 [43]	Retrospective	UK	1796			6
Wyles et al., 2019 [44]	Retrospective	USA	242	36%	72	8
Yoon et al., 2010 [45]	Retrospective	South Korea	119	5.90%	70	7

Rates of Mortality in staBTKA

**Figure 2:**
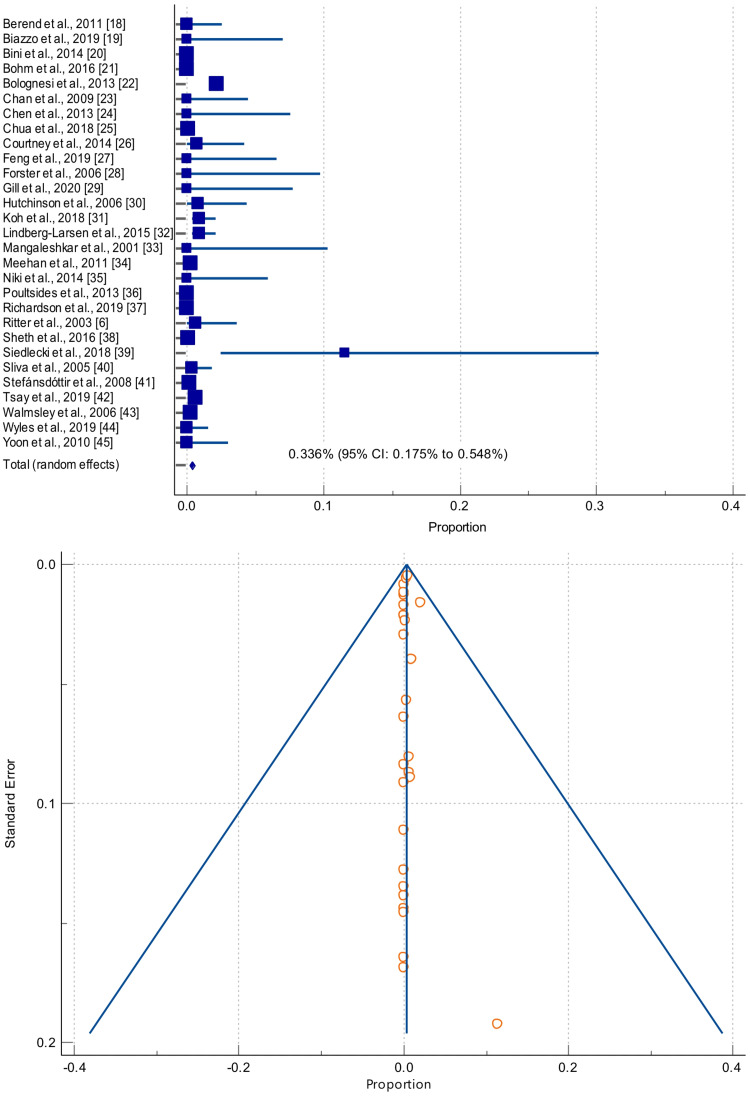
Forest plot showing the pooled mortality rate in patients who underwent staged bilateral total knee arthroplasty

A quantitative data synthesis was conducted (Table 2, Figure 2) to estimate the pooled prevalence of mortality among the patients who underwent staBTKA. The mortality rate was estimated to be 0.34% (95% confidence interval (CI): 0.18% to 0.55%). As shown in Table 3, the data showed significant interstudy heterogeneity (I^2^ = 93.3%). The highest mortality rate across the pooled data was 11.5%, as reported by a French study conducted by Siedlecki et al. [39]. We used funnel plot inspection to visually assess significant publication bias, and the plotted data showed a symmetrical distribution.

**Table 2:** Quantitative mortality data from the included studies CI: confidence interval

Study	Sample size	Proportion (%)	95% CI	Weight (%)
Berend et al., 2011 [18]	141	0.000	0.000 to 2.582	2.36
Biazzo et al., 2019 [19]	51	0.000	0.000 to 6.978	1.14
Bini et al., 2014 [20]	2132	0.000	0.000 to 0.173	5.54
Bohm et al., 2016 [21]	2123	0.000	0.000 to 0.174	5.54
Bolognesi et al., 2013 [22]	3788	2.165	1.725 to 2.680	5.78
Chan et al., 2009 [23]	80	0.000	0.000 to 4.506	1.61
Chen et al., 2013 [24]	47	0.000	0.000 to 7.549	1.07
Chua et al., 2018 [25]	12951	0.0618	0.0267 to 0.122	6.02
Courtney et al., 2014 [26]	131	0.763	0.0193 to 4.180	2.25
Feng et al., 2019 [27]	54	0.000	0.000 to 6.603	1.19
Forster et al., 2006 [28]	36	0.000	0.000 to 9.739	0.86
Gill et al., 2020 [29]	46	0.000	0.000 to 7.706	1.05
Hutchinson et al., 2006 [30]	125	0.800	0.0203 to 4.377	2.19
Koh et al., 2018 [31]	633	0.948	0.349 to 2.052	4.51
Lindberg-Larsen et al., 2015 [32]	628	0.955	0.351 to 2.068	4.50
Mangaleshkar et al., 2001 [33]	34	0.000	0.000 to 10.282	0.82
Meehan et al., 2011 [34]	26350	0.319	0.254 to 0.395	6.08
Niki et al., 2014 [35]	60	0.000	0.000 to 5.963	1.30
Poultsides et al., 2013 [36]	1151	0.000	0.000 to 0.320	5.12
Richardson et al., 2019 [37]	6110	0.000	0.000 to 0.0604	5.91
Ritter et al., 2003 [6]	152	0.658	0.0167 to 3.611	2.47
Sheth et al., 2016 [38]	7185	0.0696	0.0226 to 0.162	5.94
Siedlecki et al., 2018 [39]	26	11.538	2.446 to 30.154	0.65
Sliva et al., 2005 [40]	306	0.327	0.00827 to 1.807	3.52
Stefánsdóttir et al., 2008 [41]	3432	0.146	0.0473 to 0.340	5.75
Tsay et al., 2019 [42]	45419	0.610	0.540 to 0.686	6.10
Walmsley et al., 2006 [43]	1796	0.278	0.0905 to 0.648	5.44
Wyles et al., 2019 [44]	242	0.000	0.000 to 1.513	3.17
Yoon et al., 2010 [45]	119	0.000	0.000 to 3.052	2.12
Total (random effects)	115348	0.336	0.175 to 0.548	100.00

**Table 3:** Test for heterogeneity and publication bias CI: confidence interval; DF: degrees of freedom

Parameter	Value
Q	419.2547
DF	28
Significance level	P < 0.0001
I^2^ (inconsistency)	93.32%
95% CI for I^2^	91.46 to 94.78
Egger's test	
Intercept	-0.2766
95% CI	-2.1984 to 1.6452
Significance level	P = 0.7700
Begg's test	
Kendall's Tau	0.3300
Significance level	P = 0.0120

Discussion

The most serious consequence that may follow surgery is death, and similar to other rare complications, a large patient population must be examined to accurately determine the mortality risks associated with different treatment modalities. This meta-analysis included 29 studies with data from 115,348 patients. The pooled prevalence of mortality in TKA was 0.34% (95% CI: 0.18% to 0.55%). However, significant heterogeneity was observed in the pooled data.

The debate about whether bilateral TKA should be performed in individuals with arthritis in both knees remains unresolved. The safety of concurrent bilateral TKA is still under debate [46,47]. Intensive care unit admissions, pulmonary embolism, deep vein thrombosis, and cardiac, neurologic, and wound problems have all been linked to an increase in postoperative complications [34]. However, there is disagreement in the literature regarding whether this increases the risk of mortality [11,38,43]. Large institutional series studies have shown that simultaneous bilateral TKA may be conducted safely without an increase in perioperative morbidity and mortality compared with phased bilateral TKA [48], if a selective preoperative screening approach is implemented [49].

There is a broad range of suggested intervals between the first and second surgeries in patients undergoing staBTKA, ranging from one to 120 months [6,30,50]. Studies have shown that the indications for staBTKA and the processes used to choose the initial side for surgery differ [45,51]. While few studies have analyzed future revision rates, several comparisons have been made regarding the safety, perioperative complications, and cost-effectiveness of simBTKA versus staBTKA.

In the last 10 years, several studies have compared staBTKA and single-anesthesia BTKA. Studies with large datasets (n = 5665-407070 participants) have produced mixed results [20-22,25,34,36,38,52]. While some studies have found no differences in mortality or complication rates, including revision surgery, between simBTKA and staBTKA [20,25,36,38], others have found higher mortality rates [21,22].

## Conclusions

Death is the most catastrophic consequence that can occur after surgery, and as with other uncommon problems, a large patient group has to be analyzed in order to reliably estimate the mortality risks associated with various treatment techniques.

Our study found that the mortality rates following staBTKA are relatively high. However, the reliability of our findings is limited due to significant heterogeneity. We recommend further studies be conducted to provide higher-quality evidence to assess mortality in staBTKA and its trends.
